# Mutations of *rpoB, katG, inhA* and *ahp* genes in rifampicin and isoniazid-resistant *Mycobacterium tuberculosis* in Kyrgyz Republic

**DOI:** 10.1186/s12866-018-1168-x

**Published:** 2018-03-22

**Authors:** Jainagul Isakova, Nurmira Sovkhozova, Denis Vinnikov, Zoy Goncharova, Elnura Talaibekova, Nazira Aldasheva, Almaz Aldashev

**Affiliations:** 1 Institute of Molecular Biology and Medicine, 3 Togolok Moldo street, 720040 Bishkek, Kyrgyzstan; 2 School of Public Health, Al-Farabi Kazakh National University, Al-Farabi avenue 71, Almaty, Kazakhstan 050040; 3 National Center of Tuberculosis, 90a, Akhunbaev street, Bishkek, 720020 Kyrgyz Republic; 4 Kyrgyz-Russian Slavic University, Bishkek 44 Kievskaya street, Bishkek, 720000 Kyrgyz Republic

**Keywords:** *Mycobacterium tuberculosis*, *rpoB*, *katG*, *inhA*, *Ahp*, TB-BIOCHIP, Kyrgyz Republic

## Abstract

**Background:**

The aim of this study was to identify mutations of r*poB, katG, inhA* and *ahp*-genes associated *Mycobacterium tuberculosis* resistance to rifampicin (RIF) and isoniazid (INH) in Kyrgyz Republic. We studied 633 smear samples from the primary pulmonary tuberculosis (TB) patients. We verified *Mycobacterium tuberculosis* susceptibility to RIF and INH using culture method of absolute concentrations, and commercially available test named “TB-BIOCHIP” (Biochip-IMB, Moscow, Russian Federation).

**Results:**

For RIF-resistance, TB-BIOCHIP’s sensitivity and specificity were 88% and 97%, 84% and 95% for INH-resistance, and 90% and 97% for multi-drug resistance (MDR). In RIF-resistant strains, TB-BIOCHIP showed mutations in codons 531 (64.8%), 526 (17.3%), 516 (8.1%), 511 (5.4%), 533 (3.2%), 522 (0.6%) and 513 (0.6%) of *rpoB* gene. The most prevalent was Ser531 > Leu mutation (63.7%). 91.2% of mutations entailing resistance to INH were in *katG* gene, 7% in *inhA* gene, and 1.8% in *ahpC* gene. Ser315→Thr (88.6%) was the most prevalent mutation leading to resistance to INH.

**Conclusions:**

In Kyrgyz Republic, the most prevalent mutation in RIF-resistant strains was Ser531 → Leu in *rpoB* gene, as opposed to Ser315 → Thr in *katG* gene in INH-resistant *Mycobacterium tuberculosis*. In Kyrgyz Republic, the major reservoir of MDR *Mycobacterium tuberculosis* were strains with combined mutations Ser531 → Leu in *rpoB* gene and Ser315 → Thr in *katG* gene. TB-BIOCHIP has shown moderate sensitivity with the advantage of obtaining results in only two days.

## Background

Kyrgyzstan is a country in Central Asia with the total population of 6 million, whereas mountainous terrains with poor access to medical care occupy 90% of its territory. As reported by the WHO, Kyrgyzstan shows high incidence and mortality from TB, and the former peaked in 2001 with 167.8 cases per 100,000 population including penitentiary system patients. TB incidence in Kyrgyzstan that year was 16.8 times the worldwide threshold incidence of 10.0 per 100,000. The corresponding mortality rate that year was 27.0 per 100,000 including penitentiary system deaths. Since 2002, there was a consistent reduction in both incidence and mortality from TB in the country. By 2012, the incidence dropped by 37.8% to 104.3 per 100,000, whereas 68.1% reduction in mortality was registered, and the latter equaled 8.6 per 100,000, including penitentiary system patients. During the period of 2012–2016, both incidence and mortality continued to drop. In 2016, 93.1 new cases and 5.8 deaths per 100,000 were reported [1].

Parallel to the overall persistent reduction in TB incidence, the number of multidrug resistant (MDR) and extensive drug resistant (XDR) tuberculosis cases increased. Among all new primary smear-positive pulmonary TB cases in 2015, 26% were MDR, whereas among previously treated patients this number equaled 54.6% [1].

Treatment success in MDR TB remains low, reaching only 47–62.7% [2]. MDR TB is not only a serious clinical and epidemiological problem, but also entails substantial economic costs of management. Thus, treating patients with resistance to the main anti-TB, such as rifampicin (RIF) and isoniazid (INH), may be times more expensive compared to treatment costs incurred by the management of TB susceptible to the main medications panel.

Quite much is known about the mechanisms of resistance to RIF and INH. Thus, *Mycobacterium tuberculosis* resistance to RIF can be explained by mutations in *rpoB* gene, whereas *katG, inhA, ahpC, kasA and ndh* genes mutations are associated with INH resistance [3, 4]. Presentations from around the world report varying geographic distribution of selected mutations of this kind [5], when the most prevalent strains exhibit mutations in codons 531, 526 and 516. In countries with poor epidemiological situation around TB, including former Soviet countries, strains with mutation in codon 531 (Ser531→Leu) are the most prevalent (50–70%) ones [6, 7]. On the contrary, this codon mutation in other countries does not exceed 30–48% [8–10].

We have previously addressed and published the outcomes of our studies addressing genes r*poB, katG, inhA*, and *ahp* mutations [11, 12]. The current analysis presents data on these genes from a larger *Mycobacterium tuberculosis* DNA sample, as well as the most and the least prevalent RIF- and INH-resistant *Mycobacterium tuberculosis* strains in Kyrgyz Republic. This updated analysis also includes TB-BIOCHIP specificity and sensitivity data in detecting RIF- and INH-resistant/susceptible *Mycobacterium tuberculosis* in comparison with a standard culture method.

Therefore, local distribution of drug resistance found in circulating strains should be described based on the molecular studies. In Kyrgyzstan, mutations of *rpoB, katG, inhA*, and *ahpC* genes have not been studied in local *Mycobacterium tuberculosis* strains in detail, and major mutations leading to *Mycobacteria* resistance to RIF and INH have yet to be identified. Therefore, the aim of this study was to describe mutations in *rpoB, katG, inhA* and *ahpС* genes and to select the dominating population of RIF and INH-resistant *Mycobacterium tuberculosis* strains in TB patients living in Kyrgyzstan.

## Methods

### Patients and study design

The study commenced in 2009 and ended in 2011. Patients were recruited from the various inpatient departments of the National Centre of Tuberculosis (NCT), located in Bishkek. Six hundred and thirty-three samples of *Mycobacterium tuberculosis* were isolated from 633 patients from eight provinces of Kyrgyz Republic, including 192 from Bishkek‚ 150 from Chuy, 51 from Issyk-Kul, 66 from Naryn, 36 from Talas, 36 from Osh‚ 69 from Jalalabat, and 33 from Batken.

Tuberculosis diagnosis in recruited patients was confirmed by microscopy and culturing. We considered the following inclusion criteria: TB diagnosed for the first time in life, smear positive microscopic analysis of sputum, age 18 to 70, inpatient treatment and negative human immunodeficiency virus (HIV) test.

All patients underwent chest X-ray, smear microscopy, supplemented by the bacteriological verification of *Mycobacterium tuberculosis* at the NCT in Bishkek. This study protocol was approved by the local committee on bioethics of the NCT and the Institute of Molecular Biology and Medicine (Bishkek). All patients in this study provided written informed consent to participate.

In Kyrgyz Republic, on average, there were 1500 new cases of smear-positive TB within the period described in the manuscript. We, therefore, consider the sample size of this study (*N* = 633) sufficient for generalization of the molecular and genetic background of TB identified in Kyrgyzstan.

### Data collection

Sputum samples were collected from patients using conventional methods on the first day of admission to the clinic. Following decontamination, each sputum specimen was divided into two parts. The first portion was used to test *Mycobacterium* resistance to RIF and INH using classical phenotypic method, whereas the second portion was used to test resistance to RIF and INH using genetic approach, i.e. TB-BIOCHIP.

### *Bacteriological study and* phenotypic *drug susceptibility testing* (DSTs)

We used World Health Organization (WHO) recommendations for agent culturing in Lowenstein-Jensen culture medium and first line drug resistance testing via absolute concentrations method [13]. In Kyrgyz Republic, along with the conventional culture method of absolute concentrations with its results available 2–3 months after, they also use automatic «Bactec MGIT 960» test with liquid medium, which shortens result anticipation period to 2–3 weeks.

Whenever we detected the growth of *Mycobacterium tuberculosis,* Kyrgyz national TB guidelines mandate DSTs using the absolute concentration method on Löwenstein-Jensen medium in the NCT. The corresponding INH and RIF concentrations were 0.2 μg/mL and 40 μg/mL.

### *Molecular and genetic methods of testing resistance to* RIF and INH

Drug resistance to both RIF and INH was tested using commercially available “TB-BIOCHIP” set (JSC «BIOCHIP-IMB», Moscow, Russian Federation), which was developed by the Institute of molecular biology named after V.A. Engelhard of the Russian Academy of Science (Moscow, Russian Federation). Testing *Mycobacterium tuberculosis* resistance to RIF and INH using TB-BIOCHIP included the stages of clinical specimen decontamination, germ lysis, two consecutive stage of multiplex PCR, PCR-product (amplicon) hybridization on a biologic microchip, registration, and interpretation. All these stages are described in the product manual [13].

### Сlinical specimen decontamination and germ lysis

We used N-acetyl L-cysteine and 3% NaOH solution to decontaminate sputum specimens for 40 min at room temperature. We then resuspended cell extracts in TE buffer and centrifuged them for 10 min, and then exposed the pellet to 30 μl of lyses buffer, which contained 1% TritonX-100, and then incubated it for 20 min at 95 °C. After that we cooled the extract and centrifuged it for 10 min. Two μl aliquots of supernatant were used for polymerase chain reaction (PCR).

In order to check for possible contamination‚ we also tested negative control sets of all reagents except target DNA along with the samples in every set of amplification reactions.

### Two stages of multiplex PCR

The first PCR stage was aimed to amplify: а) nucleotide sequence of element IS6110 (309 b.p.), specific for *Mycobacterium tuberculosis,* and b) *rpoB* gene (212 b.p.), *katG* gene (166 b.p.), *inhA* gene (133 b.p.) and *ahpС* gene (126 b.p.) fragments, entailing resistance to RIF and INH. The second PCR stage of asymmetric type, aimed at collecting primarily one-chain PCR-products using amplicons from the first stage with simultaneous insertion of fluorescent marker. The second PCR stage was accomplished with primers specific for *rроВ* gene (126 b.p.), *katG* gene (140 b.p.), *inhA* gene (93 b.p.), and *ahpC* gene (96 b.p.) fragments, as well as IS6110 (110 b.p.). We used primers produced by JSC Biochip-IMB, Moscow, Russian Federation for both the first and second PCR stages [14–16].

### On-chip hybridization and microchip analysis

The corresponding procedure has been described elsewhere in detail [16].

### Hybridization outcomes registration

We tested mutations in *rpoB, katG, inhA*, and *ahpС* genes using the fluorescence intensity pattern analysis on the chip with Chipdetector portable fluorescence analyzer. This device works with Imageware software (BIOCHIP-IMB, Moscow, Russian Federation). TB-BIOCHIP can identify mutations in *rроВ* gene (27 types), *katG* (11 types), *inhA* (5 types) and *ahpС* (5 types), which result in *Mycobacterium tuberculosis* resistance to RIF and INH.

DNA extraction from clinical samples‚ PCR amplification of the *rpoB, katG, inhA* and *ahpС* genes‚ on-chip hybridization and detection of mutations were carried out by the Institute of Molecular Biology and Medicine‚ Bishkek‚ Kyrgyz Republic.

### Statistical methods

We compared phenotypic and genetic methods of detecting *Mycobacterium tuberculosis* resistance and susceptibility to RIF and INH using 2*2 tables. We calculated TB-BIOCHIP sensitivity (ability to detect true resistance) and specificity (ability to detect true sensitivity) using the formulae: specificity = Tn/(Tn + Fp) × 100%, sensitivity = Tp/(Tp + Fn) × 100%, where Tp is a true positive outcome, Tn is a true negative outcome, Fp is a false positive outcome, and Fn is a false negative outcome [17].

## Results

Patients’ age ranged from 18 to 70 years, of whom 60% were patients in the age group below 40. In total, there were 386 males (61%) and 247 (39%) females, with the corresponding male to female ratio 1.5:1.

### Identification of RIF-resistant and susceptible Mycobacterium tuberculosis using phenotypic DST and biochip

Cultural method yielded 209 RIF-resistant specimens out of the overall number *N* = 633, whereas 424 specimens were susceptible to RIF. Biochip analysis confirmed resistance to RIF in 185 (88%) specimens out of 209, whereas RIF-susceptibility was confirmed in 411 (97%) specimens out of 424. Therefore, TB-BIOCHIP resulted in 88% sensitivity and 97% specificity. The overall agreement of two methods in RIF-resistant/susceptible strains identification was 94% (596 out of 633) (Table 1).

**Table 1:** 2*2 table indicating agreement of phenotypic DST with TB-BIOCHIP in RIF-resistant/susceptible strains identification

TB-BIOCHIP	Phenotypic DST		Total
	RIF-resistant	RIF-susceptible	
RIF-resistant	185	13	198
RIF-susceptible	24	411	435
Total	209	424	633

Thirty seven (5.8%) specimens out of 633 did not match. These discrepancies can be explained with likely mutations in other *rpoB* gene loci, because TB-BIOCHIP tests only one fragment of *rpoB* gene, which is a region responsible for RIF-resistance. Mutations explaining RIF-resistance in RRDR locus (81 b.p.), can be identified in 95% *Mycobacterium tuberculosis* strains, whereas the gene itself contains 3000 base pairs [3, 4].

### Mutations in rpoB gene in RIF-resistant strains

We found 13 mutations in 7 codons of *rpoB* gene in RIF-resistant samples (*n* = 185), where the most prevalent locations were codons 531 (64.8%), 526 (17.3%), 516 (8.1%), and 511 (5.4%), whilst 513, 533 and 522 codons mutations were quite rare (Table 2), altogether explaining only 4.4% of all *rpoB* gene mutations. The commonest amino acid replacement was Ser531 → Leu in *rpoB* gene (63.7%) cases. The second leading mutation was the one in codon 526 (17.3%), which comprised five replacement variants, including His526→Tyr (4.3%), His526→Asp (5.9%), His526→Arg (5.9%), His526→Leu (0.6%, and His526→Pro (0.6%). Codon 516 was the third leading location of mutation with two variants: Asp516→Tyr (7.0%) and Asp516→Val (1.1%) (Table 2).

**Table 2:** Prevalence and mutation profile in *rpoB* gene in RIF-resistant strains in Kyrgyzstan

Gene	Codon	Amino acid replacement	Nucleotide replacement	*N* = 185 (%)	*N* (%)
*rpoB*	533	Leu533 → Pro	CTG → CCG	6 (3.2)	6 (3.2)
	531	Ser531 → Leu	TCG → TTG	118 (63.7)	120 (64.8)
		Ser531 → Trp	TCG → TGG	2 (1.1)	
	526	His526 → Tyr	CAC → TAC	8 (4.3)	32 (17.3)
		His526 → Asp	CAC → GAC	11 (5.9)	
		His526 → Leu	CAC → CTC	1 (0.6)	
		His526 → Arg	CAC → CGC	11 (5.9)	
		His526 → Pro	CAC → CCC	1 (0.6)	
	522	Ser522 → Leu	TCG → TTG	1 (0.6)	1 (0.6)
	516	Asp516 → Tyr	GAC → TAC	13 (7.0)	15 (8.1)
		Asp516 → Val	GAC → GTC	2 (1.1)	
	513	Gln 513 → Leu	CAA → CTA	1 (0.6)	1 (0.6)
	511	Leu511 → Pro	CTG → CCG	10 (5.4)	10 (5.4)

### Identification of INH-resistant and susceptible Mycobacterium tuberculosis using phenotypic DST and biochip

Cultural method yielded 135 INH-resistant specimens out of the overall number *N* = 633. Biochip analysis confirmed resistance to INH in 114 (84%) specimens out of 135. Using cultural method, 498 specimens out of 633 were INH-susceptible. Biochip test confirmed INH-susceptibility in 475 (95%) specimens out of 498. Therefore, TB-BIOCHIP resulted in 84% sensitivity and 95% specificity in detecting INH-resistant/susceptible strains. In total, 93% of INH-resistance/susceptibility outcomes matched (589 out of 633) (Table 3) when two methods were compared.

**Table 3:** 2*2 table indicating agreement of phenotypic DST with TB-BIOCHIP in INH-resistant/susceptible strains identification

TB-BIOCHIP	Phenotypic DST		Total
	INH-resistant	INH-susceptible	
INH-resistant	114	23	137
INH-susceptible	21	475	496
Total	135	498	633

Forty four (7%) specimens out of 633 did not match. These discrepancies can be explained with likely mutations in genes other than *katG, inhA* and *ahpC*, such as in *kasA* and *ndh* genes, which are not tested by the chip [3].

### Mutations in katG, inhA and ahpC genes in INH-resistant strains

As opposed to RIF, INH-resistance may be associated with mutations in a number of genes. 104 (91.2%) specimens out of 114 yielded strains with *katG* gene mutations; whereas 8 (7.0%) specimens had *inhA* gene and 2 more specimens (1.8%) had *ahpC* gene mutations (Table 3). Moreover, in five specimens of INH-resistance, we identified combined mutation of *inhA* and *ahpC* genes. Mutation in codon 315 prevailed in *katG* gene with three mutation variants, including Ser315→Thr (88.6%), Ser315→Asn (1.7%), and Ser315→Arg (0.9%).

We found nucleotide replacement in position 15 from translation initiation site of *inhA* gene (*inhA* T_15). There were also two other mutations found in positions 9 and 12 from translation initiation site in the promotor locus of *ahpC* gene (ahpC_9 and ahpC_12). In general, INH-resistant strains in Kyrgyz Republic is quite homogenous and is predominantly represented by Ser → Thr replacement in codon 315 of *katG* gene (Table 4).

**Table 4:** Prevalence and mutation profile in *katG, inhA* and *ahpC* genes in INH-resistant strains in Kyrgyzstan

Gene	Codon	Amino acid replacement	Nucleotide replacement	*N* = 114 (%)	*N* (%)
*katG*	315	Ser315→Thr	AGC → ACC	101 (88.6)	104 (91.2)
		Ser315→Asn	AGC → AAC	2 (1.7)	
		Ser315→Arg	AGC → CGC	1 (0.9)	
*inhA*	15	inhA T_15	C → T	8 (7)	8 (7)
*ahpC*	9	AhpC_9	G → A	1 (0.9)	2 (1.8)
	12	AhpC_12	C → T	1 (0.9)	

### Identification of MDR Mycobacterium tuberculosis using phenotypic DST and biochip

Cultural method revealed 174 MDR specimens out of 633, which are resistant to both RIF and INH. Biochip method confirmed MDR in 157 (90%) out of 174 (Table 5).

**Table 5:** 2*2 table indicating agreement of phenotypic DST with TB-BIOCHIP in MDR strains identification

TB-BIOCHIP	Phenotypic DST		Total
	MDR	non-MDR	
MDR	157	13	170
non-MDR	17	446	463
Total	174	459	633

Cultural method yielded 459 non-MDR specimens out of the overall number *N* = 633. TB-BIOCHIP confirmed non-MDR in 446 (97%) cases out of 459, and such susceptibility was classified as true susceptibility to RIF and INH. Therefore, TB-BIOCHIP’s sensitivity in MDR *Mycobacterium tuberculosis* identification was 90%, whereas the specificity was 97%, and the agreement rate was 95% (603 out of 633) (Table 5).

### Mutations in rpoB, katG, inhA and ahpC genes in MDR strains

MDR is a simultaneous resistance to RIF and INH, usually in *rpoB* gene leading to RIF-resistance, and *katG, inhA* or *ahpC* genes, associated with INH-resistance. 157 (25%) specimens in our study were suggestive of MDR. Table 6 shows that 86.6% (136/157) of all MDR cases was a combined mutation in *rpoB* and *katG* genes; 2.5% (4/157) cases was a combination of *rpoB* and *inhA* genes mutation, whereas combined mutation in three genes (*rpoB, katG* and *inhA*) was found in 10.9% (17/157) specimens. Dominating mutations were Ser531 → Leu replacement in *rpoB* gene combined with Ser315 → Thr replacement in *katG* gene (50.3%**)** (Table 6).

**Table 6:** Prevalence and mutation profile in *rpoB, katG, inhA* and *ahpC* genes in MDR strains in Kyrgyzstan

MDR -TB				*N* = 157	%
RIF-resistance	INH-resistance				
*rpoB*	*katG*	*inhA*	*ahpC*		
Ser531 → Leu	Ser315 → Thr	–	–	79	50.3
Ser531 → Leu	Ser315 → Thr	InhA_T15	–	3	1.9
Ser531 → Leu	Ser315 → Asn	–	–	5	3.2
Ser531 → Leu	Ser315 → Arg	–	–	3	1.9
His526 → Arg	Ser315 → Thr	–	–	7	4.5
His526 → Asp	Ser315 → Thr	–	–	6	3.8
His526 → Tyr	Ser315 → Thr	InhA_T15	–	14	9.0
His526 → Leu	Ser315 → Gly	–	–	1	0.6
His526 → Leu	–	InhA_T15	–	2	1.3
His526 → Pro	–	inhA_T15	–	1	0.6
His526 → Asn	–	inhA_T15	–	1	0.6
Asp516 → Tyr	Ser315 → Thr	–	–	13	8.3
Asp516 → Val	Ser315 → Thr	–	–	2	1.3
Leu511 → Pro	Ser315 → Thr	–	–	12	7.6
Leu533 → Pro	Ser315 → Thr	–	–	8	5.1

## Discussion

In this study, we confirmed that in TB patients living in Kyrgyz Republic, RIF-resistant *Mycobacterium tuberculosis* strains had mutations in codons 531, 526, 516, 511, 512, 513 and 533 of *rpoB* gene, whereas the most prevalent were codon 531 (64.8%) and 526 (17.3%) mutations.

In INH-resistant *Mycobacterium tuberculosis*, most common mutations occurred in *katG* gene (91.2%), *inhA* gene (7%) and *ahpC* gene (1.8%). Amongst all *katG* gene mutations, Ser315 → Thr replacement in codon 315 was likely the most prevalent. Moreover, we confirmed TB-BIOCHIP system has comparable sensitivity and specificity with culture method. Unlike the latter, TB-BIOCHIP can make drug resistance profile available for a clinician within two days, timely guiding epidemiological decision to isolate patients with MDR and correct their treatment almost immediately after the diagnosis.

The majority of RIF-resistant strains in fact have mutation in the so-called “hot” locus of *rpoB* gene (81 b.p., codon 507–533 – rifampicin resistance determining region, RRDR). *rpoB* gene codes beta-subunit of DNA-dependent RNA-polymerase which act as a major target for RIF [3, 18]. Up to 95–98% of RIF-resistant strains exhibit mutations in *rpoB* gene, whereas 90–95% of these mutations are located in RRDR [3, 18]. Despite such miscellaneous mutation locations, most of them are located in three *rpoB* codons: 531, 526 and 516.

In general, our findings of most prevalent codon 531 with Ser531→Leu (64.8%) replacement in RIF-resistant strains in Kyrgyzstan TB patients somewhat similar to other former Soviet countries and territories with high burden of MDR TB in the prevalence and the profile of *rpoB* gene mutations [14, 16, 19–24]. In general, Ser531 → Leu mutation is the prevalent among RIF-resistant strains, fund all over the world [3].

As opposed to RIF, *Mycobacterium tuberculosis* resistance to INH is associated with mutations in a number of genes. INH is a pro-drug, and its activation in a cell is performed by catalase-peroxidase, coded by *katG* gene in *Mycobacterium tuberculosis* [25]. INH activation entails a few forms of active oxygen, including superoxide, peroxide and hydroxyl radicals, nitrogen oxide isonicotinic acid radical that act at various targets in *Mycobacterium tuberculosis* cells, however enol-acidic phosphate-reductase is the principal link in mycolic acids synthesis.

INH-resistance is associated in a number of genes, including *katG* (catalase-peroxidase gene), *kasA* (β-keto-acyl-АСР-synthase gene), *ndh* (NADH-dehydrogenase), *inhA* region (NADH-dependent-ACP-reductase *InhA* gene) and *ahpC* region (alkyl-hydroxyperoxide-reductase gene). *katG* and *inhA* genes mutations are most clinically relevant and determine resistance in the most of clinical isolates [3, 26, 27].

Our TB-BIOCHIP tests showed that *katG* gene mutations were responsible for 91.2%, *inhA* gene mutations for 7%, and *ahpC* gene mutations for 1.8% of resistance to INH. Our findings are quite consistent with the world literature confirming *katG* gene Ser315 → Thr replacement to be the most prevalent (88.6%) [19, 28–31]. On the contrary, they found relatively lower *katG* gene mutations prevalence (less than 40%) in RIF-resistant strains of *Mycobacterium tuberculosis* in Tunisia [32, 33].

MDR, which is a combined resistance to RIF and INH, is a serious issue given the economic cost of treatment. Of note, spontaneous chromosome mutations leading to resistance to RIF and INH in a given population have a probability of 3.5 × 10^− 6^ and 3.1 × 10^− 8^ [3, 4], whereas the probability of MDR is times smaller and equals 9 × 10^− 14^, since responsible genes are located in different sites. This explains acceptable efficacy of this combination to treat TB in cases when the agent was initially susceptible to these medications [30]. Moreover, resistance to RIF develops the latest, therefore, RIF-resistance is a surrogate marker of MDR [30, 34, 35]. Resistance to RIF alone, however, develops exceptionally seldom, and 90% of RIF-resistance is combined with resistance to INH. Our TB-BIOCHIP tests demonstrated such pattern. Thus, 28 (15%) specimens out of 185 RIF-resistant strains were resistant to RIF only, whereas 157 (85%) cases of *rpoB* gene mutations coincided with *katG, inhA* and *ahpC* genes mutations, usually responsible for INH-resistance.

This analysis has a number of strengths. Firstly, we had an opportunity to take advantage of the use of TB-BIOCHIP method to verify mutations in *rpoB, katG, inhA* and *ahpС* gene. The method has been shown to confirm 88% RIF-resistant and more than 80% INH-resistant strains within a day. Secondly, this study, to our best knowledge, is the first attempt to describe mutations in *rpoB, katG, inhA* and *ahpС* genes in Kyrgyz Republic, which can explain MDR to TB. We should also name the opportunity to include patients and specimens from all locations around the country, including low- and high-altitude towns, as another strength of this analysis. Finally, such large sample size may be one of very few reports from this region. As for limitations, we were not able to repeat TB-BIOCHIP analysis after 2 months of treatment once cultural method results were obtained due to technical reasons.

TB-BIOCHIP has been shown to have moderate sensitivity. Its main advantage is the opportunity to obtain fast results, almost within 2 days. The timing and accuracy are essentially important for primary TB patients, when relevant treatment is pivotal to ensure treatment success and immediate segregation of patient groups in order to block the spread of resistant strains and formation of MDR. TB-BIOCHIP helps not only obtain fast results, but also enables to identify genetically opposing RIF and INH-resistant *Mycobacterium tuberculosis* variants. All these advantages justify the implementation and wide use of TB-BIOCHIP for express detection of RIF- and INH-resistant strains.

With currently moderate sensitivity, the method should be further improved and adapted to clinical practice, since missing 10% of cases in high TB/MDRTB incidence settings is challenging. Given that the biochip’s efficiency depends on the number of available cells for DNA test with at least 500 cells needed, better specimens’ collection and preparation may be the way to improve TB-BIOCHIP performance as it may alleviate DNA loss. Our study did not imply TB-BIOCHIP cost effectiveness analysis, however we consider the ability of TB-BIOCHIP to detect MDR Mycobacterium tuberculosis within 2 days as its main advantage. Saving time to the result will probably substantially contribute to reducing the cost of patient’s management.

At present, a number of molecular and genetic methods of resistance verification are either recommended or in use in various settings. WHO recommends Xpert MBT/RIF and Genotype MTBDRplus (Hain Lifescience GmbH, Nehren, Germany). Xpert MBT/RIF can verify RIF-mutations and, in fact, can only detect *Mycobacterium tuberculosis*, resistant to RIF only. Genotype MTBDRplus can confirm resistance to two medications, such as RIF and INH, as a result of only three genes mutations, including *rpoB, katG* and *inhA*. As opposed to these two options, TB-BIOCHIP can confirm resistance to both RIF and INH resulting from mutations in four genes, including *rpoB, katG, inhA* and *ahp*.

We explain the discrepancies between TB-BIOCHIP and Phenotypic DST in detecting susceptibility with a number of reasons including rare mutations, which cannot be verified with the current biochip version; mixed population in a specimen, which may comprise both resistant and susceptible strains, which may show non-homogenous growth in Lowenstein-Jensen culture medium. Moreover, not all mutations will reflect phenotypic changes when cultivating strains in a solid culture medium with set concentrations of medications. Finally, TB-BIOCHIP can detect mutations associated with RIF- and INH-mutations in both alive and dead Mycobacterium tuberculosis.

## Conclusions

In Kyrgyz Republic, Ser531 → Leu replacement in *rpoB* gene was the commonest variant of RIF-resistance, whereas Ser315 → Thr mutation in *katG* gene could likely explain most of INH-resistance using TB-BIOCHIP analysis. MDR strains in our country likely developed most of their resistance because of combined mutations Ser531 → Leu in *rpoB* gene and Ser315 → Thr in *katG* gene. Taken together, this analysis will help guide treatment of patients with resistant strains and reduce the overall burden of the disease in the country.

## Abbreviations

DNA: deoxyribonucleic acid
HIV: human immunodeficiency virus
INH: isoniazid
MDR: multi-drug-resistant
NCT: National Centre of Tuberculosis
RIF: rifampicin
USSR: Union of Soviet Socialist Republics
WHO: World Health Organization
